# Single cell model for re‐entrainment to a shifted light cycle

**DOI:** 10.1096/fj.202200478R

**Published:** 2022-09-03

**Authors:** Anouk W. van Beurden, Robin A. Schoonderwoerd, Mayke M. H. Tersteeg, Pablo de Torres Gutiérrez, Stephan Michel, Ruben Blommers, Jos H. T. Rohling, Johanna H. Meijer

**Affiliations:** ^1^ Department of Cell and Chemical Biology Leiden University Medical Center Leiden The Netherlands

**Keywords:** entrainment, PER2, phase shift, retinohypothalamic tract, suprachiasmatic nucleus

## Abstract

Our daily 24‐h rhythm is synchronized to the external light–dark cycle resulting from the Earth's daily rotation. In the mammalian brain, the suprachiasmatic nucleus (SCN) serves as the master clock and receives light‐mediated input via the retinohypothalamic tract. Abrupt changes in the timing of the light–dark cycle (e.g., due to jet lag) cause a phase shift in the circadian rhythms in the SCN. Here, we investigated the effects of a 6‐h delay in the light–dark cycle on PERIOD2::LUCIFERASE expression at the single‐cell level in mouse SCN organotypic explants. The ensemble pattern in phase shift response obtained from individual neurons in the anterior and central SCN revealed a bimodal distribution; specifically, neurons in the ventrolateral SCN responded with a rapid phase shift, while neurons in the dorsal SCN generally did not respond to the shift in the light–dark cycle. We also stimulated the hypothalamic tract in acute SCN slices to simulate light‐mediated input to the SCN; interestingly, we found similarities between the distribution and fraction of rapid shifting neurons (in response to the delay) and neurons that were excited in response to electrical stimulation. These results suggest that a subpopulation of neurons in the ventral SCN that have an excitatory response to light input, shift their clock more readily than dorsal located neurons, and initiate the SCN's entrainment to the new light–dark cycle. Thus, we propose that light‐excited neurons in the anterior and central SCN play an important role in the organism's ability to adjust to changes in the external light–dark cycle.

**Abbreviations**
ACSF: artificial cerebrospinal fluid
AIC: Akaike Information Criterion
AUC: area under the curve
LUC: Luciferase
PACAP: pituitary adenylate cyclase‐activating peptide
Per1: period 1
PER2: PERIOD2
RHT: retinohypothalamic tract
ROI: region of interest
SCN: suprachiasmatic nucleus
ZT: zeitgeber time

## 1 INTRODUCTION

All mammals have an intrinsic clock that drives their 24‐h behavioral and physiological rhythms. For our body to function optimally, it must be synchronized to the environmental light–dark cycle.^1^, ^2^ Thus, exposure to an abrupt change in the light–dark cycle—for example, jet lag due to flying to a different time zone or engaging in rotating shift work—causes a temporary misalignment between our internal clock and the environmental cycle, leading to internal desynchronization. This can result in sleep disturbances, fatigue, and loss of productivity. Occasional changes in the light–dark cycle generally cause only temporary discomfort, but repeated phase shifts in our clock can have profound negative health effects, including increased blood pressure, reduced sleep efficacy, metabolic impairment, and even a shortened life span.^3^, ^4^

The suprachiasmatic nucleus (SCN) serves as the body's master clock and is located in the hypothalamus, directly above the optic chiasm. The SCN is a relatively small bilateral structure that consists of approximately 10 000 neurons per side.^2^, ^5^ Circadian rhythms are generated autonomously at the single‐cell level via a process based on negative transcriptional and translational feedback loops between clock genes and their respective protein products.^6^, ^7^, ^8^ When the clock is stably aligned with the environmental light–dark cycle, the neurons in the SCN are synchronized near phase coherence, giving rise to a uniform rhythm in the ensemble activity, and consequently of the output.^1^, ^9^ Molecular studies have shown that an abrupt shift in the light–dark cycle causes desynchronization within the SCN due to differences in the resetting rate between the ventral SCN and dorsal SCN.^10^, ^11^, ^12^ Moreover, measurements of electrical activity have shown that the ventral SCN re‐entrains to the new light–dark cycle before the dorsal SCN.^13^ Given that even a temporary disruption of our circadian rhythm can increase the risk of certain diseases, understanding the resetting process is an important first step toward maintaining health. Therefore, we examined resetting at the single‐cell level in the SCN.

Zeitgebers are external timing cues that synchronize an organism's circadian rhythms, and light is considered the most potent zeitgeber with respect to synchronizing the SCN's clock to the external 24‐h light–dark cycle. Approximately 20%–30% of the SCN's neurons receive light‐related input from the melanopsin‐containing retinal ganglion cells via the retinohypothalamic tract (RHT).^14^, ^15^, ^16^ The fiber terminals of the RHT are most dense in the ventral SCN and less dense in the dorsomedial SCN.^17^ The RHT terminals release glutamate and—under certain conditions—pituitary adenylate cyclase‐activating peptide (PACAP).^18^ Activation of postsynaptic glutamate receptors causes an increase in intracellular Ca^2+^ concentration ([Ca^2+^]_i_) in SCN neurons, which activates the CREB signaling pathway. CREB is activated by phosphorylation and drives transcription of *Period1 (Per1*).^19^ In addition, the clock gene product Period2 (PER2) functions as mediator of light‐dependent CREB signaling^20^ and is involved in resetting the phase of the clock.^21^, ^22^ Because PER2 is an excellent marker for tracking the clock's phase,^23^, ^24^ we hypothesized that an abrupt phase delay in the light–dark cycle would induce a rapid shift in the rhythm of PER2 expression in light‐responsive neurons in the SCN. In addition, we expect that the non–light‐responsive neurons in the SCN would take longer to become synchronized with the shifted neuronal population.

To test this hypothesis, we performed ex vivo bioluminescence imaging of single‐cell PERIOD2::LUCIFERASE (PER2::LUC) gene expression to measure rhythms in the mouse SCN under a standard light–dark cycle (i.e., a fixed 12 h:12 h light–dark cycle) and after introducing a single 6‐h delay in the transition from light to dark. Previous studies showed that under normal conditions the expression of PER2 is highest at the end of the subjective day.^23^ We found that the phase shift in the PER2 rhythm induced by the delay had a bimodal distribution among the SCN neurons; specifically, one population of neurons was more shifted than the other population and comprised approximately 29% of all SCN neurons. Next, we stimulated the RHT using electrical pulses to simulate the input of light information to the SCN. The results of both experiments revealed that the population of rapid shifting neurons and the population of light‐responsive neurons largely overlap in terms of both their location and quantity. Based on these findings, the most parsimonious model is that the light‐responsive neurons readily adjust their PER2 expression pattern following a phase shift and initiate the SCN's entrainment to the new light–dark cycle.

## 2 MATERIALS AND METHODS

### 2.1 Phase delay experiments

#### 2.1.1 Animals

All animal experiments were performed in accordance with Dutch law regarding animal welfare and were approved by the Dutch government; the protocols were approved by the Animal Experiment Committee Leiden. Homozygous PER2::LUC knock‐in mice^25^ were bred at the Leiden University Medical Center animal facility.^25^ Male adult (3–7 months of age) mice were housed in climate‐controlled cabinets equipped with a full‐spectrum diffused lighting source providing 50–100 lux intensity and had access to food and water ad libitum throughout the entire experiment. All mice were housed under a 12 h:12 h light–dark (LD 12:12) cycle prior to the experiment; the time at which the lights were turned off was defined as zeitgeber time 12 (ZT12). For the mice in the delay group (*n* = 12), a 6‐h delay was introduced by delaying the time at which the lights were turned off, resulting in a single 18‐h period of light; the standard—albeit shifted by 6 h—LD 12:12 cycle was thereafter continued. For the mice in the control group (*n* = 11), the light–dark cycle was not changed throughout the entire experiment. Animals were sacrificed on the first day after the delay, 2 h before the lights were turned off, as dissection at this time was found to have the least effect on the PER2::LUC rhythm.^26^

#### 2.1.2 Bioluminescence imaging and analysis

Organotypic SCN explants were prepared as described previously.^25^ In brief, the mice were decapitated and the brain was removed and placed in ice‐cold modified (low Ca^2+^ and high Mg^2+^) artificial cerebrospinal fluid (ACSF) containing (in mM): 116.4 NaCl, 5.4 KCl, 1.0 NaH_2_PO_4_, 0.8 MgSO_4_, 1.0 CaCl_2_, 4.0 MgCl_2_, 23.8 NaHCO_3_, 15.1 D‐glucose, and 5 mg/L gentamicin (Sigma‐Aldrich, Munich, Germany) saturated with 95% O_2_–5% CO_2_ and buffered to pH 7.4. Hypothalamic slices (200 μm thick; *n* = 18 delay, *n* = 18 control) containing respectively the anterior, central and posterior SCN were prepared using a VT1000 S vibrating microtome (Leica, Microsystems, Wetzlar, Germany). The shape of the third ventricle and the thickness of the optic chiasm served as landmarks to distinguish between the anterior, central, and posterior SCN. The SCN was then excised from the slices, and each explant was placed on a Millicell membrane insert (PICMORG50, Merck‐Millipore, Burlington, MA) in a 35‐mm Petri dish containing 1.2 ml Dulbecco's Modified Eagle's Medium (D7777, Sigma‐Aldrich) supplemented with 10 mM HEPES buffer (Sigma‐Aldrich), 2% B‐27 (Gibco, Landsmeer, the Netherlands), 5 U/ml penicillin, 5 μg/ml streptomycin (0.1% penicillin–streptomycin; Sigma‐Aldrich), and 0.2 mM D‐luciferin sodium salt (Promega, Leiden, the Netherlands); the pH was adjusted to 7.2 with NaOH, and osmolality was adjusted to 295–310 mOsm.

The SCN explants were immediately transferred to a light‐tight, temperature‐controlled chamber (Life Imaging Services, Reinach, Switzerland) held at 37°C. The chamber was equipped with an upright microscope (BX51WIF, Olympus), a cooled CCD camera (ORCA–UU‐BT‐1024, Hamamatsu Photonics Europe, Herrsching am Ammersee, Germany), a motorized stage (V240 XY shifting table, Luigs & Neumann, Ratingen, Germany) and focus control (MA‐42Z, Märzhäuser, Wetzlar, Germany). Bioluminescence images of the SCN explants were obtained using an exposure time of 29 min. Image acquisition was controlled using Image‐Pro Plus software (Media Cybernetics, Warrendale PA) with the StagePro plug‐in (Objective Imaging, Cambridge, UK).

To analyze the time series of bioluminescence images, a custom‐written MATLAB‐based (MathWorks, Natick, MA) program was used as described previously by Buijink et al.^25^ In brief, a map showing peak expression in terms of luminescence intensity was created from the time series of bioluminescence images. Within the peak expression map, cell‐sized groups of 3–9 pixels with luminescence intensity higher than noise were defined as regions of interest (ROIs). The location of these ROIs had to be consistent throughout the recordings to include them for further analysis. We refer to each cell‐like ROI as a ‘single‐cell’. The average bioluminescence of all pixels in each ROI was calculated for the image series, resulting in a bioluminescence trace representing PER2::LUC expression for each single‐cell ROI. To analyze the rhythm characteristics such as peak time and period, the raw PER2::LUC expression traces were smoothed and resampled to one data point per minute. Only single‐cell traces containing at least 3 cycles with a period length of 20–28 h were used for further analysis. On average, 222 single‐cell traces (range: 113–361 traces) were included in each recording.

#### 2.1.3 Synchronization analysis

Phase distribution, period variability, and an order parameter (*R*) were calculated for all SCN explants. Phase distribution was defined as the standard deviation (SD) of the peak times of individual neurons per explant of the specified cycle in vitro. The cycle‐to‐cycle interval was determined based on the time difference between two consecutive half‐maximum values of the rising edge of the PER2::LUC expression rhythm. Period variability was defined as the SD of the cycle‐to‐cycle interval of individual cells and was calculated for the first 3 cycles for each cell in an explant and then averaged for the explant. *R* was determined by first calculating the mean peak time $\left({\bar{t}}_{p}\right)$ of PER2::LUC expression of all cells $\left(\;j=1\ldots N\right)$ for the specified cycle using the following equation:

$${\bar{t}}_{p}=\frac{\sum_{j=1}^{N}t_{p,\;j}}{N}.$$

The relative peak time for each cell was then calculated by subtracting the peak time of that cell from the mean peak time of all cells, and the relative peak time was converted to its relative phase $\left(\theta_{r}\right)$ using the following equation:

$$\theta_{r,\;j}=\frac{\left({\bar{t}}_{p}-t_{p,\;j}\right)}{\tau }\cdot 2\pi ,$$

where τ is the period in hours. The relative phase was then transformed using Euler's formula, and the absolute value was used to obtain *R* as follows:

$$R=\left|\frac{\sum_{j=1}^{N}e^{i\cdot \theta_{r,\;j}}}{N}\right|.$$

Statistical analyses were performed using SPSS version 25 (IBM, Armonk, NY). Except where indicated otherwise, groups were compared using the Mann–Whitney *U* test, and differences with a *p*‐value <.05 were considered significant.

#### 2.1.4 Single‐cell phase‐shift analysis

The phase of individual neurons is not homogeneous throughout the SCN.^25^ Thus, to calculate the phase shift of individual cells in the explants obtained from the delay group, we created spatio‐temporal maps from the single‐cell coordinates and the corresponding time of peak expression in PER2 based on the explants obtained from the control group. Template maps were generated for the anterior, central, and posterior SCN using the time of peak expression in PER2 protein in the first full cycle in vitro. First, the size of the SCN was measured in all explants, and the average value was used as the standard size for the SCN maps. Next, all control explants were aligned to the maps using coordinate transformations. Both the right and left SCN were scaled separately, and the optic chiasm and third ventricle served as landmarks to confirm accurate alignment. For overlapping cells, the average peak time of PER2 expression was calculated. There could be some gaps in the template maps due to regions in the control explants that did not contain cells. The maps were finalized by mirroring and then overlaying both halves of the SCN in order to reduce the effect of outliers and minimize the gaps in the maps. This mirroring strategy was performed because retinal input to the SCN is symmetrical, as shown by anatomical tracing studies.^5^, ^27^, ^28^ Moreover, we did not observe differences between the left and right SCN in our own data. Next, the explants from the delay group were aligned to the template maps, and the peak times from the control map were subtracted from the peak times of each individual delay explant, providing the phase shift of each individual cell. In the event when a cell in one of the delay explants was located in a gap in the template map, this resulted in the loss of information regarding this cell; this occurred in up to four cells within one explant in the delay group.

**FIGURE 1:**
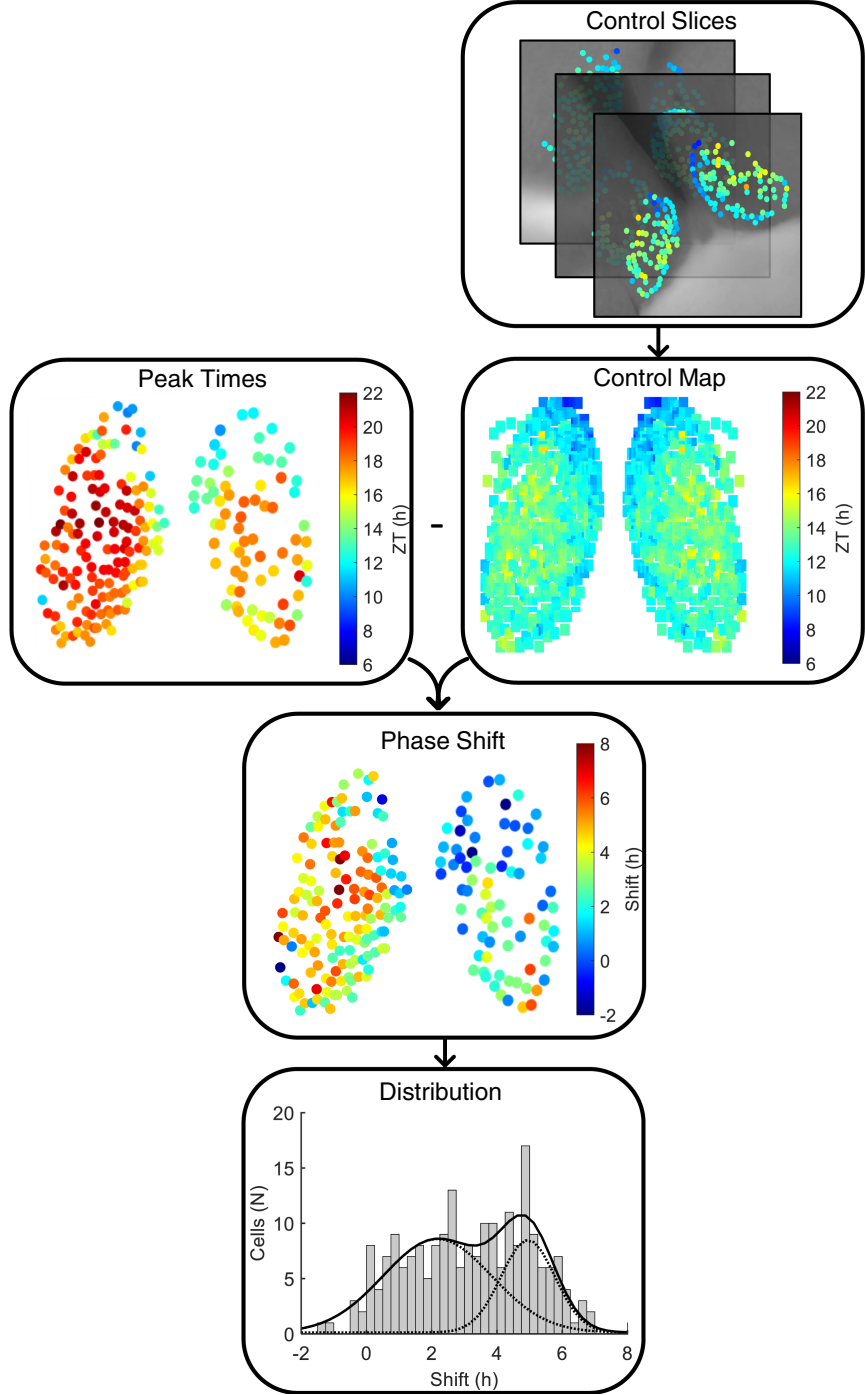
Flowchart depicting the method of phase‐shift analysis. An example of data obtained from an explant containing the anterior SCN with 218 cells is shown. First, a spatiotemporal map was created from the anterior explants in the control group. Next, the peak times from the control map were subtracted from the peak times of the delay explant to obtain the phase shift of the individual cells. Finally, the phase‐shift results were binned, and Gaussian curves were fitted to the distribution

To evaluate the distribution of the phase of cellular oscillations, histograms were created from the singe‐cell phase shifts obtained from mice in the experimental condition. The bin size was optimized and set to 15 min. Curve fitting algorithms available in Igor (http://www.wavemetrics.com) were then applied to the data to fit a curve to the histograms. Both a Gaussian function with one component and a Gaussian function with two components were fitted to each histogram. The Gaussian function describing the components was characterized as follows:

$$y=\frac{A}{w}\sqrt{\left(\pi /2\right)}e^{-2{\left(\frac{x-x_{c}}{w}\right)}^{2}},$$

where *A* represents the area under the Gaussian curve, *w* represents the width of the Gaussian curve, and *x*_*c*_ represents the time of the peak of the Gaussian curve. The resulting Gaussian function that best described the pattern of the phase shift was selected using the lowest chi‐square test statistic and the lowest Akaike Information Criterion (AIC) value. The Gaussian function with two components was considered a significantly better fit when the AIC value was at least 2 points lower than the AIC value for the Gaussian function with one component; however, when the second component was located entirely within the first component, the Gaussian function with one component was selected as having the best fit. The workflow of the phase shift analysis is illustrated in Figure 1. To calculate the individual contribution of one component, we used the area under the Gaussian curves and to localize both components within the SCN we used the intercept of the Gaussian curves as cut‐off point.

### 2.2 Optic chiasm stimulation experiments

#### 2.2.1 Animals

For these experiments, we used male C3H mice obtained from Envigo (Horst, the Netherlands). Mice at 1–2 months of age (*n* = 11) were housed as described above. All mice were subjected to a 12 h:12 h light–dark cycle throughout the entire experiment and sacrificed at ZT10.

#### 2.2.2 Ca^2+^ imaging and analysis

The mice were anesthetized with 4% isoflurane (Isoflutek 1000) and decapitated. The brain was dissected and then stirred for 1 min in modified ACSF to reduce neuronal activity. Acute coronal slices (300 μm thickness; *n* = 16) were cut using a VT1000 S vibrating microtome in modified ACSF and subsequently maintained in oxygenated standard ACSF containing (in mM): 116.4 NaCl, 5.4 KCl, 1.0 NaH_2_PO_4_, 0.8 MgSO_4_, 1.8 CaCl_2_, 23.8 NaHCO_3_, 15.1 D‐glucose, and 5 mg/L gentamicin saturated with 95% O_2_–5% CO_2_ (pH 7.2–7.4 and 290–310 mOsm). The slices were incubated for 20 min at 37°C, followed by 1 h at room temperature.

The cells were then loaded with the Ca^2+^ indicator dye Fura‐2‐AM (Fura‐2‐acetoxymethyl ester) based on a protocol described previously.^29^ In brief, the slices were incubated with Fura‐2‐AM (7.29 μM, AAT Bioquest, Sunnyvale, CA) at room temperature for 1 h and then placed in a recording chamber that was continuously perfused with oxygenated standard ACSF (at a rate of 1.5–2.0 ml/min) using a model BPS‐4 valve‐controlled gravity perfusion system (ALA Scientific Instruments, Westbury, NY). The chamber was mounted on the fixed stage of an Axioskop 2‐FS Plus upright fluorescence microscope (Carl Zeiss Microimaging, Oberkochen, Germany). A Polychrome V monochromator (TILL Photonics, Munich, Germany) was used to excite the Ca^2+^ indicator alternating between 340 nm and 380 nm light, and the emitted light (505 nm) was captured using a cooled CCD camera (PCO Imaging Sensicam, TILL Photonics) mounted on the microscope. Images were acquired every 2 s and recorded using TILLVision software (TILL Photonics). A custom‐built bipolar electrode (Pt/Ir, Fork, Teflon‐coated, 2x Ø50 μm) was placed in the optic chiasm perpendicular to the third ventricle and used to stimulate the RHT. A Grass Instruments S88 Dual Output Square Pulse Stimulator, Grass SIU5 Stimulus Isolation Unit, or Neuro Data PG4000 Digital Stimulator was used to generate trains of pulses of submaximal stimulus intensity (3–4 V, 20 Hz, 4‐ms duration for 10 s). The optimal voltage was determined experimentally before the experiment. Each Ca^2+^ recording lasted approximately 120 s and included the delivery of two trains of electrical pulses at an interval of 60 s. All recordings were performed between ZT13 and ZT18.

Images were analyzed using TILLVision software and a custom‐made analysis program written with Python version 3.0.9. For each recording, the neurons were selected as the ROIs, and a region without neurons was used as background. The average fluorescence intensity measured within each ROI for both the 340 and 380 nm channels was computed over time. The mean fluorescence intensity was then adjusted by subtracting the background for each time point and was used to calculate the Fura‐2 excitation ratio as follows:

$$r=\frac{F_{340}}{F_{380}}\;.$$

Next, the approximate Ca^2+^ concentrations for every r were calculated:

$$\left[{\mathrm{Ca}}^{2+}\right]=\beta \cdot K_{d}\cdot \frac{r-r_{\min}}{r_{\max}-r},$$

where *β* is 7.48, *K*_*d*_ (the dissociation constant) was 230 nM, and *r*_min_ and *r*_max_ (the minimum and maximum ratio values, respectively) were 149 and 5262, respectively; these constants were determined experimentally by calibration prior to the recording. Neurons with a basal [Ca^2+^]_i_ > 300 nM were excluded from the analysis. A graphical map of the [Ca^2+^]_i_ values across the experiment was obtained. In addition, we developed an algorithm to analyze the resulting [Ca^2+^]_i_ transients. Baseline values (*bl*) were calculated by averaging the [Ca^2+^]_i_ values measured over the 10 s time interval prior to application of the stimuli. To determine the transient duration (*t*_*d*_), the start of the transient was set as the start of the stimulation, and the end point was set as the timepoint at which [Ca^2+^]_i_ returned to within 10% of the corresponding baseline. Next, the area under the curve (AUC) was calculated for the transient, resulting in positive values for transient [Ca^2+^]_i_ increases and negative values for transient [Ca^2+^]_i_ decreases. In addition, we performed a qualitative analysis of the responses in order to categorize each response as an excitatory, inhibitory, or null response. For the qualitative analysis, the normalized AUC values (AUC_*n*_) were calculated by dividing by the baseline value and duration of the transient, as follows:

$${\mathrm{AUC}}_{n}=\frac{\mathrm{AUC}}{\mathrm{bl}\cdot t_{d}}.$$

Transients with an AUC_*n*_ <0.9 were classified as inhibitory responses, transients with an AUC_*n*_ >1.1 were classified as excitatory responses, and transients with an AUC_*n*_ between 0.9 and 1.1 were classified as null responses. Responses were only included in the analysis if the [Ca^2+^]_i_ transients were of the same response type for both stimulations (i.e., both were excitatory responses or both were inhibitory responses).

The localization the light‐responsive neurons, was plotted, and compared with the localization of rapidly shifting PER2 expressing cells.

## 3 RESULTS

### 3.1 Peak times of PER2::LUC expression

**FIGURE 2:**
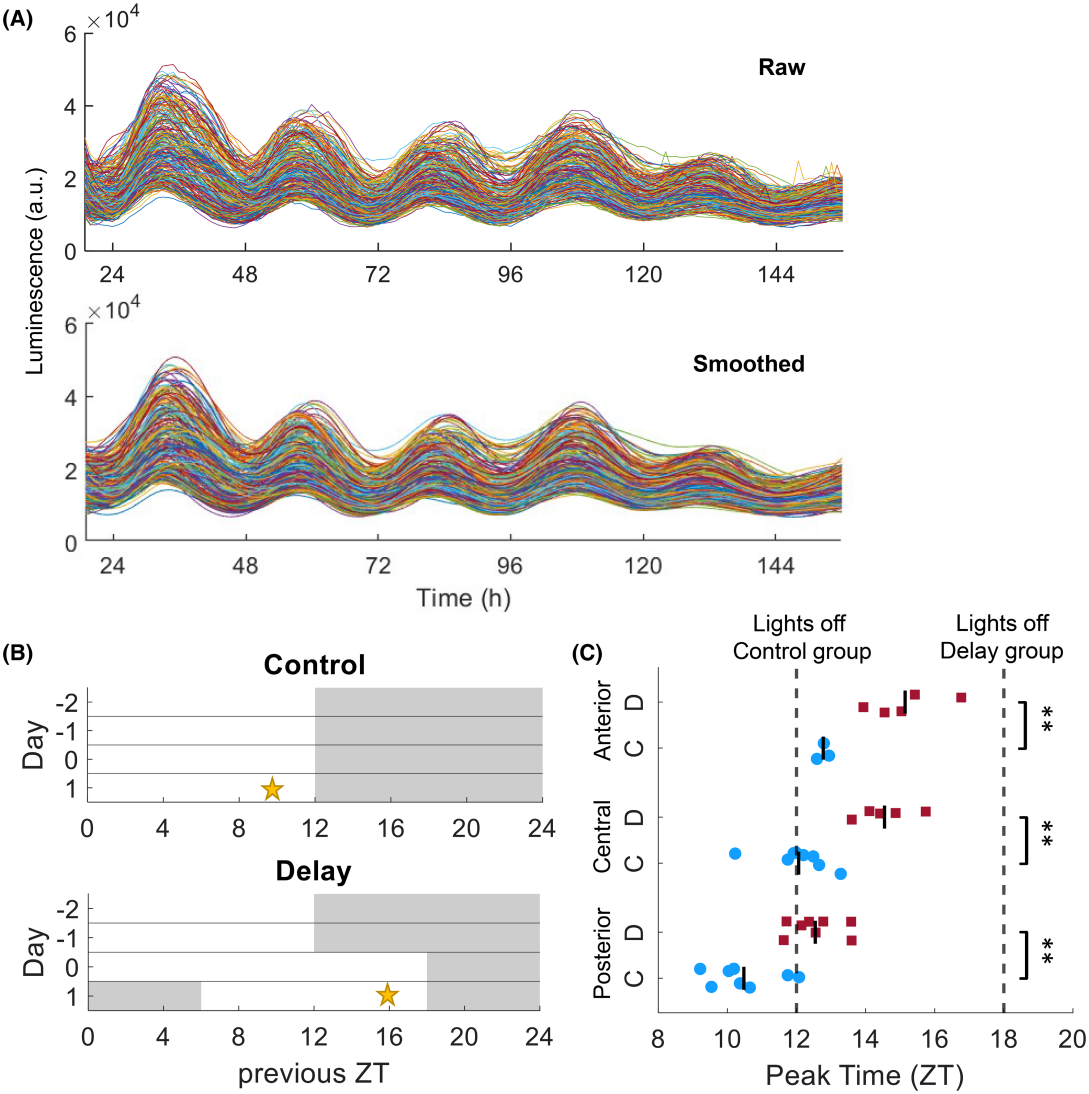
The peak time of PER2::LUC expression is shifted following a 6‐h delay in the light–dark cycle. (A) An example of raw and smoothed bioluminescence intensity traces are shown, showing PER2::LUC expression measured in individual cells in the central SCN after inducing a 6‐h delay (*n* = 265 cells). (B) Protocol for the control and delay groups. In the delay group, the lights remained on for an additional 6 h (for a total of 18 h of light); in the control group, the lights were turned off at the regular time. On day 1, the mice were sacrificed 2 h before the lights were turned off (indicated by the yellow stars at ZT10 and ZT16 for the control and delay groups, respectively. (C) Summary of the peak times of PER2::LUC expression measured on day 2 in the anterior, central, and posterior SCN in the control (“C”, solid blue circles) and delay (“D”, solid red squares) groups, plotted in zeitgeber time (ZT). The vertical lines indicate the mean values, and each symbol represents an SCN explant. The dashed lines indicate the time at which the lights were turned off in the control and delay groups. **p < .01

To determine the peak times of PER2 expression after introducing a 6‐h delay in the light–dark cycle, PER2::LUC peak times were analyzed from the averaged smoothed bioluminescence intensity traces recorded for each explant and then compared between the delay and control groups for the first full cycle in vitro. Figure 2A shows an example of the raw and smoothed bioluminescence single‐cell traces, and Figure 2B shows the protocols for the control and delay groups. On day 0 the lights remained on for an additional 6 h in the delay group. On day 1, the mice were sacrificed 2 h before the lights were turned off. The following day (i.e., day 2 relative to the delay, or after 1 cycle in vitro), the average peak time was approximately 2.5 h later in the delay group compared to the control group (Figure 2C; anterior, delay: ZT 15.1 ± 1.1, *n* = 5, control ZT 12.7 ± 0.2, *n* = 3; *p* < .01; central, delay: ZT 14.5 ± 0.8, *n* = 5, control ZT 12.0 ± 0.9, *n* = 8; *p* < .01; posterior, delay: ZT 12.5 ± 0.8, *n* = 8, control ZT 10.4 ± 1.0, *n* = 8; *p* < .01). This finding indicates that a moderate phase shift in PER2 expression occurred almost immediately after the shift in the light‐dark cycle. The data on peak time showed that the temporal relationship from the anterior SCN to the posterior SCN is maintained after a delay is introduced in the light–dark cycle.^25^ We found that the peak times did not change significantly in the anterior, central, or posterior SCN over the subsequent 3 days (Figure S1).

### 3.2 Temporal desynchronization following a phase delay

**FIGURE 3:**
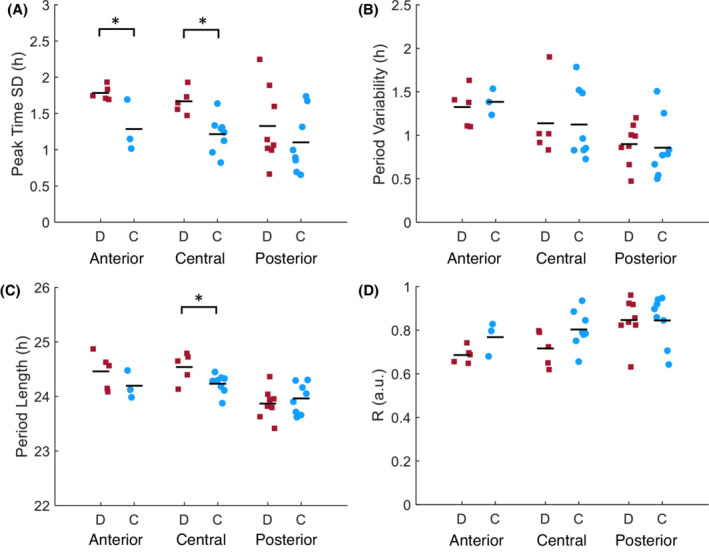
A phase delay causes desynchronization of neurons in the anterior and central SCN. (A) Phase distribution, measured as the standard deviation (SD) of the peak times in individual cells from the first full cycle in vitro (B), period variability, calculated as the SD from the differences in period length between the first 3 cycles in vitro (C), period length, calculated as the average time difference between the half‐maximum of the rising edge of the PER2::LUC expression rhythm for the first 3 cycles in vitro (D), and the order parameter (R), calculated as the relative phase difference between cells for the first full cycle in vitro; are plotted for the delay (solid red squares) and control (solid blue circles) groups. The horizontal lines indicate the mean values, and each symbol represents an SCN explant. *p < .05

To determine whether introducing a phase delay affects the synchronization of neurons within the SCN, we calculated the peak time dispersion, variability in the single‐cell period, period length, and order parameter (*R*) for each explant in the control and delay groups (Figure 3). For the peak time dispersion and order parameter, the first full cycle in vitro was used; for variability in the single‐cell period and period length, the first 3 cycles in vitro were used. In both the anterior and central SCN—but not the posterior SCN—peak time dispersion was significantly higher in the delay group compared to the control group (Figure 3A; anterior, delay: 1.8 ± 0.1 h, *n* = 5, control 1.3 ± 0.5 h, *n* = 3; *p* < .05; central, delay: 1.7 ± 0.2 h, *n* = 5, control 1.2 ± 0.2 h, *n* = 8; *p* < .05; posterior, delay: 1.3 ± 0.5 h, *n* = 8, control 1.1 ± 0.4 h, *n* = 8; *p* = .36), potentially indicating reduced synchronization in these two parts of the SCN. The effect of reduced synchronization holds‐up on the subsequent 3 cycles (result not shown). With respect to the other three parameters examined, we found no significant difference between the delay group and the control group, with the exception of period length, which was slightly longer in the central SCN of the delay group compared to the control group (Figure 3C; central, delay: 24.6 ± 0.3 h, *n* = 5, control 24.2 ± 0.2 h; *p* < .05).

### 3.3 The phase‐shift response has a spatially distinct pattern in the SCN

**FIGURE 4:**
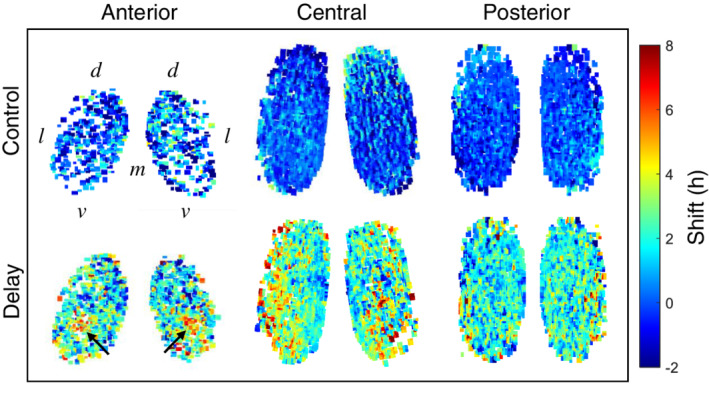
Spatial differences in the phase‐shift response in the delay group. Average spatiotemporal maps of the phase shift in PER2 expression measured in the first full cycle in vitro in the anterior, central, and posterior SCN in the control group (*n* = 3–8) and delay group (*n* = 5–8). Note the cluster of cells in the anterior SCN in the delay group with a large delay of 5–6 h (arrows). d, dorsal; l, lateral; m, medial; v, ventral

The finding that the delay increased peak time dispersion in the anterior and central SCN but not in the posterior SCN indicates that the SCN contains spatially distinct neuronal subpopulations with specific phase‐shifting responses. To examine this in further detail, we calculated the phase shift in the first full cycle in vitro for each individual cell from the explants in the control and delay groups. To evaluate the spatial pattern of the phase‐shift responses in the SCN, we averaged the spatiotemporal maps for both groups, as shown in Figure 4. We found that in the control group, the cells in the anterior, central, and posterior SCN had virtually no phase shift, which is expected given that the templates were based on the data from the control experiments.

In contrast, the cells in the delay group had a relatively large phase shift throughout the SCN. Interestingly, we found a small cluster of cells in the ventral region of the anterior SCN with a shift of approximately 5–6 h, consistent with the 6‐h delay in the light–dark cycle. We also found a gradual decrease in this phase shift in the direction of the dorsal part of the anterior SCN. In the central SCN, the largest phase shift occurred in the ventrolateral region and decreased in the direction of the dorsomedial region. Finally, in the posterior SCN we found that the majority of cells had a relatively small phase shift of 2–4 h, with no clear spatial pattern.

### 3.4 The phase shift induces a bimodal phase distribution

**FIGURE 5:**
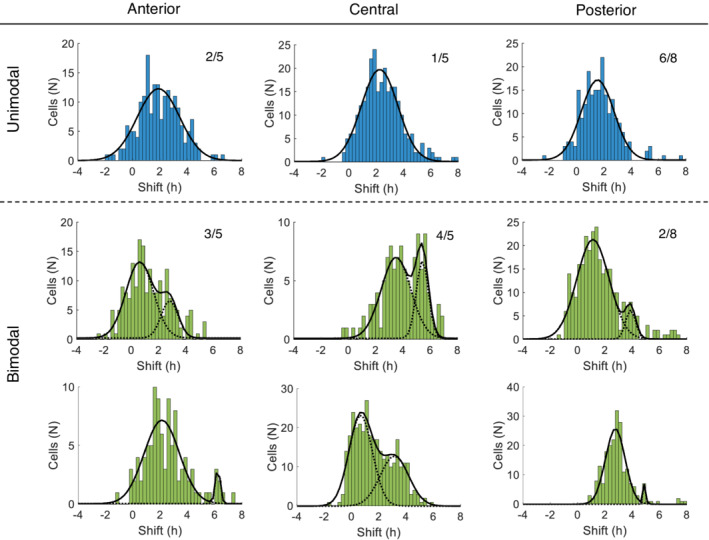
Representative examples of unimodal and bimodal phase distributions of SCN neurons in the delay groups. Each histogram represents the distribution of the phase shifts of all individual neurons within a single explant for the first full cycle in vitro, plotted in 15‐min bins. The phase shift (in hours) is plotted on the x‐axis, and the number of cells is plotted on the y‐axis. The data were fit to either unimodal or bimodal Gaussian functions. In each plot, the proportion of explants in which a unimodal or bimodal distribution was identified is provided

To analyze the phase‐shifting responses of the explants, we generated distribution histograms of the observed shift with 15‐min bins. For each explant, we created a histogram containing the phase shift measured for all neurons in the first full cycle in vitro; we then fit Gaussian curves to each histogram in order to analyze the phase distribution; whether a unimodal or bimodal Gaussian curve resulted in a better fit was based upon the AIC value. Representative examples of both unimodal and bimodal phase distributions in the delayed explants are shown in Figure 5. We found a bimodal distribution in 60% (3/5) of the anterior explants, 80% (4/5) of the central explants, and 25% (2/8) of the posterior explants.

**FIGURE 6:**
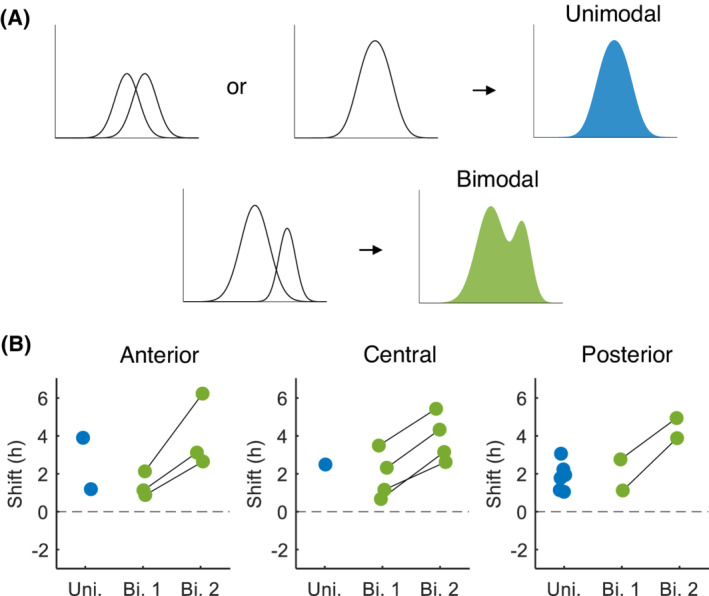
Summary of the phase shift for the unimodal and bimodal neuronal subpopulations. (A) Idealized examples of situations in which the ensemble distribution of subpopulations results in a unimodal (blue, top) or bimodal (green, bottom) distribution. (B) For delay explants with a unimodal phase distribution (Uni., blue dots) the average phase shift in PER2 expression is shown for the first full cycle in vitro for anterior, central, and posterior SCN and for explants with a bimodal distribution the average phase shift of the first (Bi. 1) and second (Bi. 2) population (green dots) are shown

The average phase shift within an explant is summarized in Figure 6. For the explants with a bimodal distribution in phase shift, the average phase shift of the first and second population are presented separately. The average phase shift in the delayed explants was 2.3 h and ranged from 0.7 h (in the first population) to 6.2 h (in the second population). A Wilcoxon signed‐rank test revealed a significant difference in phase shift between the two populations (Pop. 1: 1.7 ± 0.9 h, Pop. 2: 4.0 ± 1.3 h, *n* = 9, *p* < .01). The area under the Gaussian curves was then used to determine the relative contribution of both populations to the phase shift (Table 1). The first and second populations consisted on average of 71% and 29% of the neurons, respectively. The contribution of the second population was smaller in the posterior SCN (~6% of the neurons), as compared to the anterior and central SCN.

**TABLE 1:** Relative contributions of the first and second subpopulations to the bimodal phase‐shift pattern

	Shift Pop. 1 (h)	Shift Pop. 2 (h)	Cells Pop. 1 (%)	Cells Pop. 2 (%)
Anterior (*n* = 3)	0.88	2.65	63.0	37.0
	2.13	6.23	94.4	5.6
	1.13	3.12	26.5	73.5
Average	1.38	4.00	61.3	38.7
Central (*n* = 4)	2.32	4.33	59.2	40.8
	1.26	2.62	38.9	61.1
	3.49	5.43	71.6	28.4
	0.67	3.15	59.7	40.3
Average	1.94	3.88	57.4	42.6
Posterior (*n* = 2)	2.76	4.94	96.2	3.8
	1.12	3.88	91.7	8.3
Average	1.94	4.41	94.0	6.0
Total average^a^	1.75	4.10	70.9	29.1

^a^ The total average is corrected for the number of anterior, central, and posterior SCN explants.

### 3.5 Stimulation of the RHT primarily causes excitation in responding neurons in the ventral SCN

**FIGURE 7:**
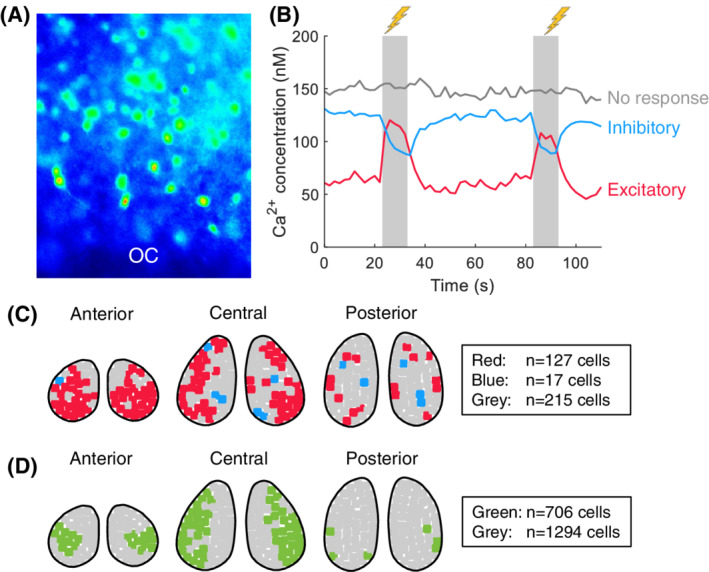
Stimulating the RHT causes a Ca^2+^ response in the SCN. (A) Example image of an SCN slice loaded with the Ca^2+^ indicator Fura‐2; the approximate location of the optic chiasm (OC) is indicated. (B) Representative traces of [Ca^2+^]_i_ measured in three SCN neurons; the RHT was stimulated where indicated by the shaded areas. One neuron responded with an increase in [Ca^2+^]_i_ (the red trace), one neuron responded with a decrease in [Ca^2+^]_i_ (the blue trace), and the third neuron did not respond (the gray trace). (C) Schematic depiction of the approximate location of excitatory (red), inhibitory (blue), and non‐responsive (gray) neuronal populations in the anterior, central, and posterior SCN based on the average of 2, 8, and 6 anterior, central, and posterior slices, respectively (see also Table 2). (D) Schematic depiction of the approximate location of rapid shifted (green) and non‐shifted (gray) neuronal populations in the anterior, central, and posterior SCN of mice in the delay group (based on the average of 3, 4 and 2 anterior, central and posterior explants with a bimodal phase shift distribution, respectively)

We found a clear bimodal distribution in the phase‐shift response in half (9 out of 18) of the delayed explants. To test whether the population of these rapid shifting neurons is the same as the light‐responsive neurons and is located within the same SCN region, we loaded SCN slices with the Ca^2+^ dye Fura‐2 and then electrically stimulated the optic chiasm to activate the RHT in order to simulate the input of light information to the SCN,^30^ while recording [Ca^2+^]_i_. An example of a Fura‐2‐AM–loaded slice is shown in Figure 7A. We found that the stimulation‐induced [Ca^2+^]_i_ signals measured in the SCN could be classified as excitatory (reflected by a reproducible transient increase in [Ca^2+^]_i_), inhibitory (reflected by a reproducible transient decrease in [Ca^2+^]_i_), or absent (no response); examples of all three responses are shown in Figure 7B. Our analysis revealed that 62%, 3%, and 35% of cells in the anterior SCN were excitatory, inhibitory, and non‐responsive, respectively. In the central SCN, 37%, 5%, and 58% of cells were excitatory, inhibitory, and non‐responsive, respectively; finally, in the posterior SCN 18%, 3%, and 79% of cells were excitatory, inhibitory, and non‐responsive, respectively. Figure 7C shows a schematic representation of the approximate locations of the three responses in the anterior, central, and posterior SCN, and the results are summarized in Table 2. Note that while the anterior and posterior regions are clearly defined in the corresponding slices, some of the slices containing the central SCN may have contained small parts of anterior and/or posterior SCN. An independent‐samples median test revealed a significant difference in excitatory responses between the anterior, central, and posterior SCN (*p* < .05).

**TABLE 2:** Summary of the responses measured in SCN neurons upon electrical stimulation of the RHT

Region	Total cells (*n*)	Excitatory response, *n* (%)	Inhibitory response, *n* (%)	No response, *n* (%)
Anterior SCN (*n* = 2 slices)	30	20 (66.7)	1 (3.3)	9 (30)
Central SCN (*n* = 8 slices)	207	90 (43.5)	11 (5.3)	106 (51.2)
Posterior SCN (*n* = 6 slices)	122	17 (13.9)	5 (4.1)	100 (82)
Total	359	127 (35.4%)	17 (4.7%)	215 (59.9%)

For comparison, we also created a schematic representation of the approximate locations of the rapid responding SCN neurons in the delay group (Figure 7D). Comparing the data in Figure 7C,D shows there is considerable overlap between the stimulus‐excited neuronal populations (shown in red in Figure 7C) and the rapid shifting neuronal populations (shown in green in Figure 7D) in terms of both quantity and location in the anterior and central SCN.

## 4 DISCUSSION

Following a shift in the light–dark cycle, it can take several cycles for the clock to readjust to the new light–dark cycle.^12^ Here, we investigated the phase‐shift response in the SCN at the single‐cell level after introducing a 6‐h delay in the light–dark cycle. We found a broadened phase distribution in PER2 expression in the anterior and central SCN in mice that experienced the delay compared to control mice. This broadened phase distribution was likely caused by one subpopulation of neurons that responded rapid to the shift in the light cycle and one with a slow phase transition. Calculating the phase shift in each neuron revealed a bimodal ensemble distribution in most of the SCN explants, and our detailed analysis of explants with a bimodal phase distribution after a shift in the light‐dark cycle revealed spatial overlap between the neurons that responded rapidly to the delay and the neurons that had an excitatory response to RHT stimulation. These results support a model in which light‐excited neurons are the same neurons that shift their PER2 rhythm following a shifted cycle.

The distinction between rapid and slower shifting neurons was particularly evident in the anterior and central SCN, resulting in bimodal ensemble patterns in 7 out of 10 explants. Importantly, we found that the neurons that responded rapidly to the shift in the light–dark cycle were located primarily in the ventrolateral part of the SCN. This result is in agreement with previous studies that investigated the molecular expression of clock genes at the population level.^10^, ^11^, ^12^ In those studies a bimodal pattern in PER2 expression was observed in a jet‐lag protocol. This is the first study to show a bimodal phase‐shift distribution in PER2 gene expression at the single‐cell level following a shift in the light–dark cycle.

Current models of photoentrainment suggest that the SCN's clock shifts in response to environmental light via the excitatory action of glutamate.^19^, ^31^ Consistent with this notion, we found a correlation between the neurons that shifted rapidly in the delay group and the neurons that showed an excitatory response to electrical stimulation of the RHT. PER2 expression is a robust marker of the clock's phase,^23^, ^24^ as the PER2 protein is part of the molecular feedback loop involved in rhythm generation. Our finding that the peak in PER2 expression in 29% of the SCN neurons shifts rapidly in response to a delay in the light–dark transition is consistent with the role of retinorecipient SCN neurons. Although this putative link has not been made explicitly in previous studies, it fits nicely with the current models of photoentrainment in which glutamate signaling and changes in intracellular Ca^2+^ lead to a shift in the molecular cycle.^19^, ^31^

In the posterior SCN, we found relatively fewer rapidly shifting neurons, and we rarely found a bimodal pattern at the cell‐population level. When we did find a bimodal distribution in the phase‐shift response, the rapid responding cells were only a small fraction (<10%) of cells. If—as we hypothesized—retinorecipient neurons shift rapidly in response to a phase delay, this finding may be explained by the relatively small percentage of neurons with an excitatory response to RHT stimulation (approximately 18% of cells) in the posterior SCN. This small population of rapidly shifting neurons may also explain why we did not find a broadened phase distribution in PER2 expression in the posterior SCN in the delay group (i.e., their contribution to the overall pattern may have been too low). Taken together, these findings are consistent with the previous finding that the RHT does not innervate the posterior SCN to the same extent as the anterior and central SCN.^32^

In some explants, we observed a unimodal distribution in the phase‐shift responses following a phase delay; however, this finding does not necessarily suggest that the underlying resetting kinetics differ from explants with a bimodal distribution. We previously investigated how the activity patterns of two subpopulations can lead to either a unimodal or bimodal ensemble distribution and found that a bimodal ensemble distribution only occurs when specific conditions are met.^33^ After introducing a delay in the light–dark cycle, a bimodal ensemble distribution can occur if the number of rapid responding neurons is relatively small compared to the number of slow responding neurons and if the phase distribution of the rapid responding neurons is relatively narrow. Even if two groups of oscillators are present, a unimodal ensemble distribution is more likely to occur. Thus, we propose that the unimodal distributions in our data may also reflect two underlying components.

In our RHT stimulation experiments, we found that overall approximately 60% of neurons did not respond with any change in [Ca^2+^]_i_. Moreover, the percentage of non‐responding neurons was larger (~80%) in the posterior SCN than in the anterior and central SCN (~30%–50%). These non‐responding neurons in the SCN are likely not directly connected to the RHT^34^; however, another possible explanation is that some of the RHT fibers may have been cut when preparing the slices.

The percentage of neurons with an inhibitory response was quite low, averaging approximately 5% throughout the anterior, central, and posterior SCN. In our companion paper, we report that inhibitory responses to light are due to the release of GABA (γ‐aminobutyric acid) directly from RHT fibers.^35^ The location of inhibitory neurons was indistinguishable from the excitatory neurons; however, the percentage of inhibitory neurons was too small to allow any speculation regarding their role in entrainment. Nevertheless, the resulting inhibition corresponds to a decrease in intracellular Ca^2+^, which would counteract the proposed signaling route for photic entrainment.^36^

Our results contribute to the current models regarding photoentrainment. It is generally accepted that photoentrainment occurs via Ca^2+^‐mediated transcription of *Per1*.^19^, ^31^ Moreover, PER2 plays a role in CREB‐mediated *Per1* induction^20^ and is induced by external light on a relatively slow timescale.^37^ The excitatory activity of SCN neurons drives the opening of AMPA and NMDA channels causing a transient increase in [Ca^2+^]_i_ as a second messenger.^19^ Previous studies have shown that approximately 20–30% of SCN neurons receive light‐mediated input directly from the RHT.^14^, ^15^, ^16^ Here, based on our measurements performed at the single‐cells level, we show that: (1) approximately 30% of neurons in the SCN undergo a rapid phase shift in response to a delay in the light–dark cycle, (2) these neurons are located primarily in the ventrolateral part of the SCN, and (3) these neurons overlap spatially with neurons that respond with excitation to RHT stimulation. Taken together and within the framework of a model for photic entrainment, we now propose that light‐excited retinorecipient SCN cells undergo a rapid phase shift following a delay in the light–dark cycle. This proposition accords with the general view that depolarization leads to a phase shift,^19^, ^31^ but narrows it down to the depolarization in the retinorecipient cells. This model is testable and may guide future experiments.

## AUTHOR CONTRIBUTIONS

Johanna H. Meijer, Jos H.T. Rohling, Anouk W. van Beurden, and Stephan Michel designed the study. Mayke M.H. Tersteeg, Robin. A. Schoonderwoerd, Pablo de Torres Gutiérrez, Stephan Michel, and Ruben Blommers performed the experiments. Anouk W. van Beurden, Jos H.T. Rohling, Robin A. Schoonderwoerd, Pablo de Torres Gutiérrez, Stephan Michel, and Ruben Blommers analyzed the data. Anouk W. van Beurden, Robin A. Schoonderwoerd, Jos H.T. Rohling, and Johanna H. Meijer wrote the paper with input from all other authors, and all authors reviewed and approved the paper.

## FUNDING INFORMATION

This research was supported by an ERC Advanced Grant (project number 834513 to J.H.M.).

## DISCLOSURES

The authors declare no competing interests.

## Supporting information


Text S1

Dataset S1

Dataset S2

Dataset S3

Dataset S4

Dataset S5

Dataset S6

Dataset S7

Dataset S8

Dataset S9

Dataset S10

Dataset S11

Dataset S12

Dataset S13

Dataset S14

Dataset S15

Dataset S16

Dataset S17

Dataset S18

Dataset S19

Dataset S20

Dataset S21

Dataset S22

Dataset S23

Dataset S24

Dataset S25

Dataset S26

Dataset S27

Dataset S28

Dataset S29

Dataset S30

Dataset S31

Dataset S32

Dataset S33

Dataset S34

Dataset S35

Dataset S36

Dataset S37

## Data Availability

The data that support the findings of this study are available in the methods and/or Supporting Information of this article.
