# Supplementary material for: Single cell model for re‐entrainment to a shifted light cycle
Source: FASEB J. 2022 Sep 3;36(10):e22518. doi: 10.1096/fj.202200478R (PMC9543151; doi:10.1096/fj.202200478R)
Supplement: Supplementary file 1 — Text S1 [file FSB2-36-0-s015.pdf]

This folder contains the data of the jet lag experiments. Each excel file holds all cells recorded in one experiment. The mice were entrained to a 12-h light 12-h dark photoperiod. The mice in the delay group were subjected to a 6 hours phase delay. The tabs contain the following data:

Information: cell number and location (X and Y) of the cell within the SCN.

Raw Data: raw time traces of all the cells. The Raw Data has one data point per hour. The upper row holds the cell numbers.

Raw Time: time points at which measurements are done. The time points are also given in Zeitgeber Time and External Time.

Smoothed Data: resampled time traces of the raw data. The smoothed data has one data point per minute. The upper row holds the cell numbers.

Analysis: contains the peak time, trough time, rising edge, falling edge and period of each cell for each cycle.

<u>Delay Anterior:</u> 191008-1, 191104-1, 200113-1,200316-1, 200608-1

<u>Delay Central:</u> 200106-2, 200113-2,200120-1, 200316-2, 200608-2

<u>Delay Posterior:</u> 191014-2, 191028-1, 191104-2, 191111-1, 191125-2, 200106-1, 200120-2, 200720-2

<u>Control Anterior:</u> 200428-1, 200622-2, 200824-2

<u>Control Central:</u> 200203-1, 200224-1, 200414-1, 200420-1, 200622-1, 200706-2, 200824-1, 200831-2

<u>Control Posterior:</u> 200203-2, 200224-2, 200302-1, 200420-2, 200428-2, 200629-2, 200706-1, 200831-1

The following cells were excluded from the experiments:

191008 – slice 1: 185
191014 – slice 2: 39
191028 – slice 1: 26
191104 – slice 2: 345
191125 – slice 2: 3, 37, 44, 67, 115, 170, 201, 209, 210
200113 – slice 2: 265
200120 – slice 2: 9
200224 – slice 2: 82, 86
200316 – slice 1: 45, 141
200608 – slice 1: 117, 128
200622 – slice 2: 153
200831 – slice 1: 22, 73
200831 – slice 2: 155
