# Supplementary material for: Single cell model for re‐entrainment to a shifted light cycle
Source: FASEB J. 2022 Sep 3;36(10):e22518. doi: 10.1096/fj.202200478R (PMC9543151; doi:10.1096/fj.202200478R)
Supplement: Supplementary file 39 [file FSB2-36-0-s009.pdf]

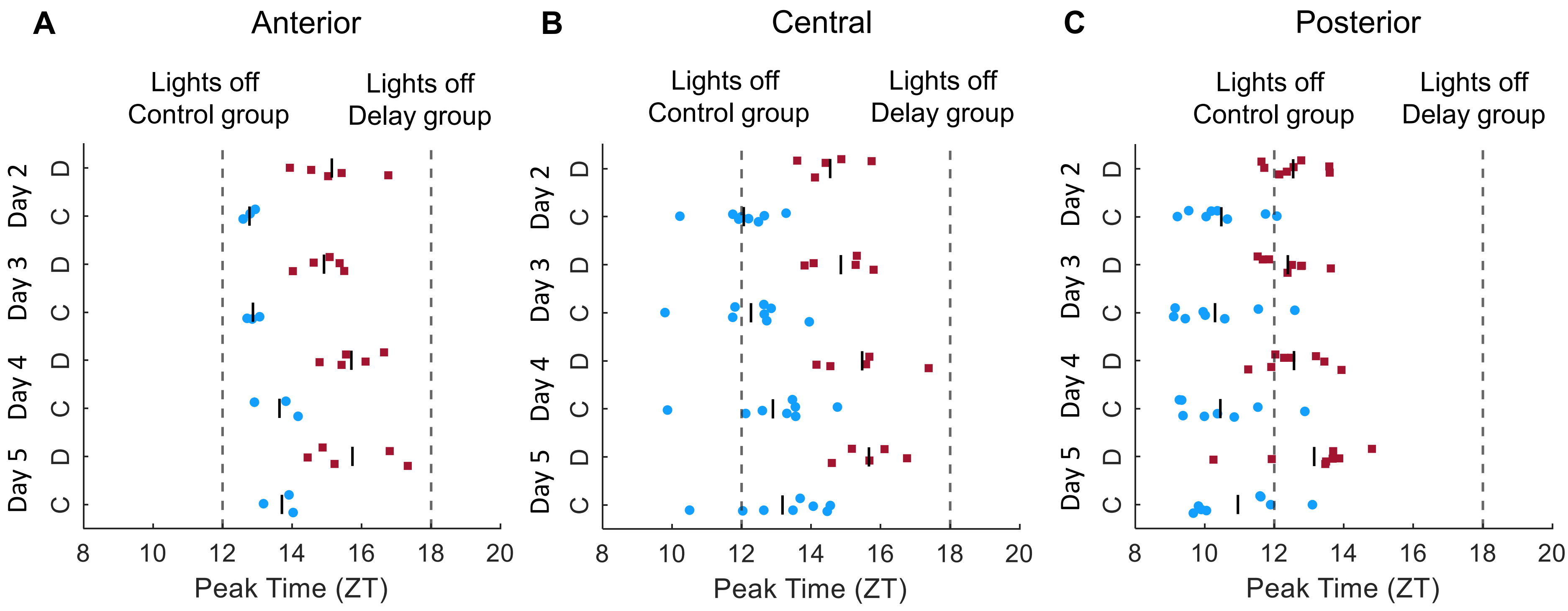

Supplemental Figure S1: Peak times in PER2::LUC expression in the SCN of control mice and mice subjected to a 6-hour delay in the light-dark cycle (see Fig. 2B). *Averaged peak times of PER2::LUC expression are plotted for each explant containing the* ***(A)*** *anterior SCN,* ***(B)*** *central SCN, and* ***(C)*** *posterior SCN in the control ("C", solid blue circles) and delay ("D", solid red squares) groups, plotted in zeitgeber time (ZT). The vertical lines indicate the mean values, and each symbol represents an SCN explant. The dashed lines indicate the time at which the lights were turned off in the control and delay groups.*
